# Clinical Trial Registration: The Differing Views of Industry, the WHO, and the Ottawa Group

**DOI:** 10.1371/journal.pmed.0020378

**Published:** 2005-10-18

**Authors:** Karmela Krleža-Jerić

## Abstract

Registration of clinical trials is an essential step in ensuring that data from all trials are reported. However, there are differing opinions on what registration should include.

In its effort to develop worldwide standards of trial registration, the World Health Organization (WHO) has formally launched its International Clinical Trials Registry Platform (ICTRP) project. The project is “taking the lead in setting international norms and standards for trial registration and reporting” [1].

As part of the project, on 25–27 April 2005, the WHO organized a technical consultation on clinical trial registration standards. The outcome of this consultation included a definition of the type of trials that need to be registered, and also of a 20 item “minimal dataset” (outlining 20 characteristics of the trial) that needs to be registered. The project has, in addition, decided to establish a unique identifier scheme [1], and to develop a worldwide search portal for trials.

Unfortunately, the pharmaceutical industry arrived at that meeting with an already established position that five items in the minimal dataset are often commercially sensitive. Hence, for some of its trials, the industry proposed to either delay these items' public disclosure or not make them publicly available at all. It proposed instead to allow them to be held in escrow, to be seen by medical journal editors at the time of manuscript evaluation, or just shown to the WHO [2].

However, even the proposed minimal dataset leaves out many of the crucial items in the Ottawa Statement on principles for trial registration (http://ottawagroup.ohri.ca/statement.html) published by the Ottawa Group, which consists of over 100 individuals and organizations worldwide (http://ottawagroup.ohri.ca/signatories.html).

In this article, I briefly consider the origins of the global movement for trial registration, and then compare the three different approaches taken by the pharmaceutical industry, the ICTRP, and the Ottawa Group (Table 1).

**Table 1:**
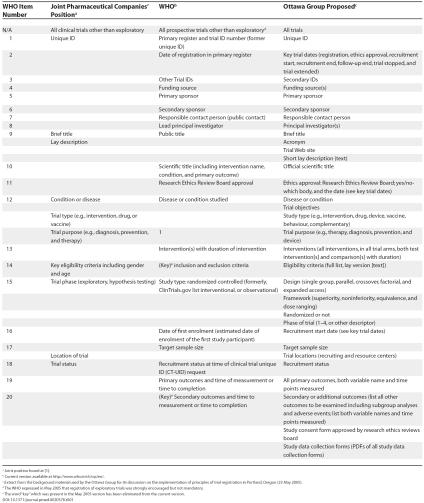
Comparison of Trial Registration Protocol Items Proposed by the Pharmaceutical Industry, the WHO, and the Ottawa Group

## Why We Need Trial Registration

The need for trial registration has been recognized for years. However, its urgency became particularly apparent in 2004 with the revelation that trial data on the harms of specific serotonin reuptake inhibitors in children went undisclosed [3]. The importance of registering trials has been made clear, for example, by the attorney general of New York's legal action against GlaxoSmithKline [4], and by the decision of medical journal editors (including the editors of *PLoS Medicine* and *PLoS Clinical Trials*) not to publish any trial unless it is has been registered [5–7].

Transparency in research and knowledge sharing are now widely seen as a precondition for the success of the health research enterprise. Trial registries are an important tool to help achieve this transparency. Just as research methodology evolved due to advances in technology, so the Internet has made electronic registries entirely feasible. The vision of freely accessible electronic depositories of protocol information of ongoing trials and their results, with each trial assigned a unique number so that it can be tracked, is now achievable.

## Trial Registries

Many registries have been developed to meet various specific needs. Here I will briefly present two that have been important in the discussions and decision-making processes of the WHO, the Ottawa Group, and the International Committee of Medical Journal Editors (ICMJE). These registries are the International Standard Randomized Controlled Trial Number (ISRCTN) register, available at http://www.controlled-trials.com/isrctn/, and ClinicalTrials.gov, run by the National Library of Medicine (NLM) at http://www.clinicaltrials.gov/. They both have accumulated practical experience by existing for more than five years. Although starting from different points, and having some different specific goals, they overlap on the important registration premise to provide free electronic access to deposited, essential information of ongoing trials. In summary, ISRCTN was created between 1998 and 2000 by a working group of researchers, mainly systematic reviewers, organized by the publisher Current Science Group (http://www.controlled-trials.com/information/faqs.asp), whereas ClinicalTrials.gov was formed five years ago by the NLM to meet United States legal requirements, and, as required by the US Food and Drug Administration (FDA) or US law, to enable a portal to register drug-related trials in the area of serious and life-threatening disease, and to enable potential participants to identify such studies in order to participate in them. The US National Institutes of Health, through NLM, has developed this Web site in collaboration with the FDA as a result of the FDA Modernization Act, which was passed into law in November 1997 [8,9]. Since 2004, and in response to recent intensive international dialogue, each registry has been changing as needed in various ways: expanding their fields, becoming publicly owned, or accepting registration of all trials.

## The Importance of Knowledge Sharing

Unfortunately, most current ongoing trials are not built on *all* existing knowledge because all knowledge is not made publicly available—it remains hidden in different pockets by various interested entities. This lack of knowledge sharing is an obstacle to the further creation of knowledge. Even when (some) clinical trial information is shared, the sharing is often delayed until a drug is on the market or, in a somewhat better case, until the trial is published. It typically takes 10–15 years between the initial idea for a trial—formulating and testing the idea, developing a trial protocol, peer reviewing, gaining funding and ethics approval, implementing the trial, developing the consequent intervention (including a drug)—and making it available to those who need it. Thus, we might have to wait up to 15 years to learn about a given trial. However, if the trial is never published, or if further studying is abandoned for whatever reason, this information has a high chance of never being shared.

Such a delay in disclosing ongoing trials means that several different research teams may be studying the same thing simultaneously in isolation, and, hence, are not building on the existing evidence but rather working on a part of the currently available evidence. Precious time and resources are wasted. More importantly, trial participants may be unnecessarily exposed to risk if the substance studied has harmful effects that have not (yet) been disclosed. Above all, research on humans can only be justified if the knowledge arising from that research is made publicly available for the public good. Only by registering trials prospectively can we be sure that all trials that are undertaken are also reported.

## International Interest in Trial Registration: The Ottawa Group

Given the importance of knowledge sharing, an international group of those interested in trial registration, including systematic reviewers and pharmaceutical industry representatives, began a dialogue on trial registration in Ottawa, Canada, during the Cochrane Colloquium in October 2004 [10]. This meeting was initiated by the Canadian Institutes of Health Research—a neutral but interested party that has been registering the trials it funds [11].

The aim of the Ottawa Group dialogue is to discuss and reach global consensus on trial registration, and to understand the meaning of registration for the group members' professional lives, for research, and for medicine and health in general.

The October 2004 meeting led to part I of the Ottawa Statement [10], summarized in Box 1. The statement has also been translated into and published in Japanese [12], and other translations are under way. The principles in this statement are simple and clear: disclose the protocol details of all trials up front, disclose amendments along the way, and post the results at the end (http://ottawagroup.ohri.ca/statement.html). Part I was used in the WHO-led worldwide consensus building while a dialogue continues, and part II of the statement, which discusses implementation principles, will follow.

**Box 1. Principles Outlined in Part 1 of the Ottawa Statement**

- Objective
- Definitions
- Rationale for international trial registration
- Types of trials to be registered
- Elements of registration
- Principles relating to unique ID, protocol registration, and registration of trial results
- Organization and language of registries
- Responsibilities of involved parties

## The Pharmaceutical Industry's Response

The Ottawa Statement has received endorsements from over 100 individuals and groups on all five continents, but so far there has not been a single signatory from a drug company (the list of signatories is available at http://ottawagroup.ohri.ca/signatories.html). This failure of endorsement by the pharmaceutical industry was unexpected since the Ottawa Statement proposes principles of trial registration, and its endorsement does not mean that any company would need to start implementing these principles immediately and begin registering all details of all trials right away.

The closest that anyone from the pharmaceutical industry has come to endorsing the principles of trial registration are Jesse A. Berlin (Johnson and Johnson) in his commentary, “Why Industry Should Register and Disclose Results of Clinical Studies—Perspective of a Recovering Academic” [13]. While Ronald Krall and Frank Rockhold in their letter, “GSK Has Created Useful Register” [14], hinted at such a possibility, in a subsequent letter by Krall and Rockhold as a reaction to the publication of part I of the Ottawa Statement, they stated that while “we reiterate our support for the principle of transparency,” the authors believed, “there is reason for concern about the amount of detail called for by the Statement” [15]. Both letters reflect clearly the January 2005 pharmaceutical industry statement [2], reconfirmed again in September 2005 [16], and mostly concentrate on the industry's concern about the disclosure of protocol details of early, so-called hypothesis-creating trials.

Most current ongoing trials are not built on *all* existing knowledge.

Although this lack of endorsement by the industry might imply that the Ottawa principles are reaching too far, this is not the case. For example, the minimum set of data items proposed by the Ottawa Statement is already on the electronic National Institute of Health/NLM ClinicalTrials.gov register, with which the industry is familiar. Rather, this lack of endorsement by the industry explains the fields left empty or filled in with meaningless information by certain pharmaceutical companies in these registries (D. Zarin [Director of ClinicalTrials.gov], personal communication; [17]). Using Zarin's analysis in May 2005, the ICMJE stated, “Many entries in the publicly accessible ClinicalTrials.gov database do not provide meaningful information in some key data fields,” and demanded the validation of registration data [17].

## The WHO ICTRP Minimal Dataset

Several members of the Ottawa Group were among many participants at the WHO technical consultation on clinical trial registration standards in April 2005. Other participants included medical journal editors, pharmaceutical companies, and the two major registries, ClinicalTrials.gov and the ISRCTN register.

A major outcome of this consultation was the previously mentioned 20 item minimal dataset for trial registration. This dataset has formally been supported by the ICMJE [17].

However, as mentioned, full up front disclosure of five of these 20 key items is being challenged by the pharmaceutical industry [18]. The five items that industry disagrees with the automatic public disclosure of are the official scientific title of the study (item 10), the intervention (item 13), the target sample size (item 17), the primary outcome (item 19), and the key secondary outcomes (item 20). The industry has agreed, instead, to place these five hidden items for an unknown proportion of trials in escrow—that is, into an electronic depository that the public cannot access or will only be able to access with a delay. Table 1 gives a comparison of the minimum registration data laid out by the WHO ICTRP project and the pharmaceutical industry, and proposed by the Ottawa Group.

This comparison of the ICTRP and Ottawa statements shows that the requirements of the ICTRP statement are less far reaching. The ICTRP statement seems to reflect the industry's concerns in that the wording of 15 out of 20 items agreed on by the WHO ICTRP project in April 2005 is similar to those agreed on by pharmaceutical companies in its January 2005 industry statement [1,2]. The document arising from the WHO technical consultation also states that “one or more of data items 10, 13, 17, 19, 20 may be regarded as sensitive for competitive reasons by the sponsor who may wish to delay release of the information” [18]. However, the WHO's current proposal is that to get registered and obtain a unique identification (ID), all 20 items must be provided to the WHO. Although the ICTRP statement is a great beginning to this WHO project, which aims to be a compromise between the differing positions on public disclosure, the Ottawa Statement should be seen as the goal to which we should aspire.

## Why Are All Twenty Items Essential to Understand a Trial?

The pharmaceutical industry claims [1,16] that a disclosure of one or all five data items would endanger proprietary rights, or as Krall and Rockhold put it, “jeopardize the competitive investment which underlines the creation of new medicines” [15]. But in reality, the lack of public availability of the five key data items raises many problematic issues—for example, it would need to be clear who would approve the reason for withholding a data item from the public or the decision about the eventual timing of disclosure.

The twenty items complement each other, and arguably provide the very minimum dataset needed to understand an ongoing trial. One of these items is the unique ID, nine are administrative, and ten are trial descriptors, the disclosure of five of which are being challenged by the industry. This leaves us with only five trial descriptors as follows: brief title, condition, key inclusion criteria, study type, and recruitment status. Looking at these five items, it becomes clear that they alone are not sufficient to provide meaningful information about any given trial, and would make trial registration meaningless to the public; i.e., although these data may have been provided to the register and made available to editors, if they are not publicly available, it will be impossible for anyone else to understand the trial.

Table 2 illustrates what the description of a trial might look like with the five disputed trial descriptors omitted. For clarity, I also omitted some administrative data items (Table 2). Evidently, we do not know very much about this trial.

**Table 2:**
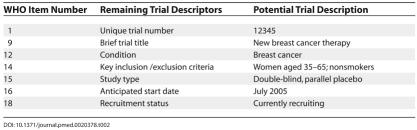
An Imaginary Breast Cancer Study with Only the Remaining Trial Descriptors

Trial registration has multiple functions, from a registry being a recruitment tool to being a means to fulfill legal obligations (I have already mentioned the FDA requirement of registration in case of serious and life-threatening diseases) to broad knowledge sharing, i.e., full disclosure. By mutually complementing each other, full public disclosure of the proposed WHO 20-item minimal registry set would meet all three main functions; without this, registration will not fulfill some of these functions. So although, as pointed out by the pharmaceutical industry [19], these 15 remaining items might seem sufficient for recruitment of trial participants, they do not meet scientific and ethical criteria; hence, all 20 are essential for trial registration.

## Conclusion

Knowledge sharing is crucial to health research, and trial registration is an indispensable part of such sharing. It is important that standards for universal trial registration be defined and applied globally.

At present, there are clearly major differences among various interested parties concerning which trial characteristics must be made publicly available at registration. As these different parties are all partners and stakeholders in the clinical trials research enterprise, they need to find a compromise between proprietary interests and knowledge sharing. I believe that new ways will have to be developed to protect intellectual and commercial interests and rights, without harming the public interest. There will have to be a change in attitudes to deal with this issue. We have indicated this need for change leading to a new paradigm of health research based on transparency, full disclosure, and collaboration in our earlier paper [10]. We have to keep in mind that although protection of commercial interests is important, the social contract with trial participants must surely take precedence.

As these compromises are worked out, journal editors stand firm in their position of requiring a precise trial registration, following the WHO–ICRTP standards. In its effort to move the initiative forward following the April 2005 meeting, the WHO issued the invitation to review and provide comments on three outstanding items: clarification of the WHO Registration Data Set (the “20 items”); criteria for register certification; and the unique ID assignment process, all of which are available in the “open comments” section on its Web site, available at http://www.who.int/ictrp.

The Ottawa Group is also continuing its worldwide dialogue on principles of operationalization of trial registration, and it will continue to contribute to the global, intergovernmental efforts of the WHO. The group holds its next face-to-face meeting in Melbourne, Australia, during the Cochrane Colloquium on 24 October 2005.

## Abbreviations

FDA: Food and Drug Administration
ICMJE: International Committee of Medical Journal Editors
ICTRP: International Clinical Trials Registry Platform
ID: identification
NLM: National Library of Medicine
WHO: World Health Organization
